# Acute Succinate Administration Increases Oxidative Phosphorylation and Skeletal Muscle Explosive Strength via SUCNR1

**DOI:** 10.3389/fvets.2021.808863

**Published:** 2022-01-14

**Authors:** Guli Xu, Yexian Yuan, Pei Luo, Jinping Yang, Jingjing Zhou, Canjun Zhu, Qingyan Jiang, Gang Shu

**Affiliations:** Guangdong Laboratory for Lingnan Modern Agriculture and Guangdong Province Key Laboratory of Animal Nutritional Regulation, National Engineering Research Center for Breeding Swine Industry, College of Animal Science, South China Agricultural University, Guangzhou, China

**Keywords:** succinate, SUCNR1, oxidative phosphorylation, muscle explosive strength, mitochondrion

## Abstract

Endurance training and explosive strength training, with different contraction protein and energy metabolism adaptation in skeletal muscle, are both beneficial for physical function and quality of life. Our previous study found that chronic succinate feeding enhanced the endurance exercise of mice by inducing skeletal muscle fiber-type transformation. The purpose of this study is to investigate the effect of acute succinate administration on skeletal muscle explosive strength and its potential mechanism. Succinate was injected to mature mice to explore the acute effect of succinate on skeletal muscle explosive strength. And C2C12 cells were used to verify the short-term effect of succinate on oxidative phosphorylation. Then the cells interfered with succinate receptor 1 (SUCNR1) siRNA, and the SUCNR1-GKO mouse model was used for verifying the role of SUCNR1 in succinate-induced muscle metabolism and expression and explosive strength. The results showed that acute injection of succinate remarkably improved the explosive strength in mice and also decreased the ratio of nicotinamide adenine dinucleotide (NADH) to NAD^+^ and increased the mitochondrial complex enzyme activity and creatine kinase (CK) activity in skeletal muscle tissue. Similarly, treatment of C2C12 cells with succinate revealed that succinate significantly enhanced oxidative phosphorylation with increased adenosine triphosphate (ATP) content, CK, and the activities of mitochondrial complex I and complex II, but with decreased lactate content, reactive oxygen species (ROS) content, and NADH/NAD^+^ ratio. Moreover, the succinate's effects on oxidative phosphorylation were blocked in SUCNR1-KD cells and SUCNR1-KO mice. In addition, succinate-induced explosive strength was also abolished by SUCNR1 knockout. All the results indicate that acute succinate administration increases oxidative phosphorylation and skeletal muscle explosive strength in a SUCNR1-dependent manner.

## Introduction

Skeletal muscle function is a crucial prerequisite to ensure quality of life, in physical exercise, and in athletic sports. Based on muscle contraction intensity and time, endurance exercise, and resistance exercise require different contraction units and energy metabolism patterns (1). Endurance exercises are mainly dependent on the aerobic metabolism of glucose and fatty acid (2). Acetyl-CoA produced from glycolysis of glucose, oxidation of pyruvate, and β-oxidation of fatty acid produces nicotinamide adenine dinucleotide (NADH) by tricarboxylic acid (TCA) cycle to generate adenosine triphosphate (ATP) (3). The latter relies on the glycolysis of glucose and glycogen to produce ATP rapidly with a byproduct-lactate accumulation (4). Therefore, individuals with stronger endurance exercise usually have weaker explosive power, and vice versa.

In recent years, a series of studies have shown that a variety of energy metabolism intermediates, such as α-ketoglutarate (AKG) and fumarate, can be used as important molecule signals to regulate cell metabolism by inducing autocrine or paracrine (5–7). As an intermediate of the TCA cycle, chronic treatment of cardiomyocytes with 1 mM succinate (SUC) will activate the hypertrophic signaling pathway through SUC receptor 1 (SUCNR1) (8). And short-term treatment with extracellular SUC leads to mitochondrial fission by SUCNR1 in the membrane of cardiomyocytes (9). Previous studies also demonstrated that long-term administration of SUC could convert fast muscle fiber into slow muscle fiber, which improves endurance exercise ability (10, 11). However, whether SUC has any effect on short-term explosive strength is not clear.

SUC has been considered as an important metabolic signal molecule with diverse potential mechanisms. Similarly, mitochondrial SUC is the substrate of complex II and produces fumarate with its oxidation (12). Extracellular SUC could not be permeated across mammalian cell membranes (11, 13, 14) but could be recognized by SUCNR1, which has been revealed to mediate the effects of extracellular SUC on several biological processes, such as adipose tissue thermogenesis (15), mitochondrial biogenesis (11), and mitophagy (16). However, there is still controversy about whether this receptor is expressed in the cell membrane of skeletal muscle myotube (17, 18). And the role of SUCNR1 in regulating the energy metabolism of skeletal muscle remains unclear.

In this study, we conducted both *in vivo* and *in vitro* experiments and revealed that acute SUC administration increased the explosive strength of muscle and energy production increased by oxidative phosphorylation. We also found SUCNR1 mRNA and protein are expressed in skeletal muscle and C2C12 myotube. Both SUCNR1-KD cell models and SUCNR1-knockdown mouse models confirmed that SUCNR1 mediated the effects of SUC on oxidative phosphorylation and skeletal muscle explosive strength.

## Materials and Methods

### Animal Experiments

All animal breeding and experiments comply with ‘the instructive notions with respect to caring for laboratory animals' issued by the Ministry of Science and Technology of the People's Republic of China; the ethics number of our experiment is 2021b121. C57BL/6J mice (3 weeks old) from the Medical Experimental Animal Center of Guangdong Province (Guangzhou, Guangdong, China) were housed in a 12-h light–dark cycle room under conditions of controlled room temperature (23°C ± 3°C) and humidity (70 ± 10%). After 6 weeks of age, healthy male mice weighing 22–26 g were randomly divided into different groups according to their body weight. All mice were fasted for 1 h, and muscle force was detected as standard; after 0.5 h to rest, the muscle force was tested again at a different time of acute injection to varying concentrations of SUC. After injection, mice were sacrificed and their eyeballs collected to get blood samples. Then the serum, biceps, gastrocnemius, soleus, tibialis anterior, and extensor digitorum were collected. The SUCNR1-knockout mouse in this study was designed by the Shanghai Model Organisms Center (Shanghai, China), and the details have been introduced in a previous study (10). Briefly, sgRNAs of SUCNR1 were knocked into zygotes *in vitro* to delete exon 2 of SUCNR1 by clustering regularly, interspaced short palindromic repeats CRISPR-associated proteins 9 (CRISPR-Cas9) systems, and the embryo was transferred to pseudo-pregnant recipients to obtain F0 generation. Male and female heterozygous mice were mated to produce the homozygous SUCNR1 mice and the wild-type (WT) mice in the same cages. The gene types of next-generation mice were identified by PCR.

### Cell Culture

The C2C12 cell line (ATCC) was cultured in a growth medium (high-glucose DME/F-12 medium with 10% fetal bovine serum) in a Thermo Scientific CO_2_ incubator (37°C, 95% humidity, 5% CO_2_). When cells grew to 80%, they were inoculated into plates uniformly. The density of these C2C12 cells was 3 × 10^4^/cm^2^. The culture medium was switched to the differentiated medium (high-glucose DME/F-12 medium with 2% horse serum) after cells reached 90% to induce differentiation. After being induced for 4–6 days, the mature muscle fibers were observed, and the differentiated cells were treated with SUA for 1 h and then collected for further detection.

### SUCNR1 siRNA Transfection

When C2C12 cells were grown to 60%, instructions for the use of Lipofectamine 2000 (Invitrogen, Carlsbad, CA, USA) were followed to transfect SUCNR1 siRNA to cells. Suzhou GenePharma Co., Ltd (Suzhou, China) designed the siRNA sequences: 5′-GCUUCUACUACAAGAUTT-3′ and 5′-AUCUUGUAGUAGAAGCTT-3′. After transfected cells were grown to 80%, cells were inoculated and treated as mentioned before.

### Immunofluorescence

Muscles were sliced into 10 μm by a cryostat (CM1850, Leica), after fixing in Tissue-Tek OCT. Muscle slices were fixed with 4% paraformaldehyde for 10 min, washed three times per 5 min by PBS, and permeabilized with Triton X-100 (0.5%, 10 min), washed three times per 5 min by PBS. Obtained sections were blocked with blocking buffer (5% BSA and 5% normal goat serum) for 30 min and then incubated with primary antibodies overnight. Primary antibodies were mouse anti-MyHC I (BA-D5-S, 1:200, DSHB) and rabbit anti-SUCNR1 (NBP1-00861, 1:1,000, Novus). Sections were washed in PBS three times per 10 min and incubated with Cy3-AffiniPure Goat-Anti-Rabbit IgG (H+L) (111-165-045, UNIV) and Alexa Fluor 488-AffiniPure Goat-Anti-Rabbit IgG (H+L) (115-545-003, UNIV), followed by secondary antibodies (1:2,000, 1 h) and then washed (three times, 10 min/time). The slides were observed with a Nikon ECLIPSE Ti microscope.

### Protein Expression Analysis

Protein was extracted from C2C12 cells with RIPA lysis buffer (P0013B, Beyotime), and the concentration of protein was detected by a BCA protein assay kit (23227, Thermo Scientific). The concentration of all proteins was adjusted to 1 μg/μl and denatured with a protein sample loading buffer (LT 101, Epizyme). Western blotting (WB) was performed as described in the previous study (19). Primary antibodies anti-SUCNR1 (NBP1-00861, 1:1,000, Novus) and tubulin (AP0064, 1:5,000, BioWord) were used. Protein signals were visualized by Protein Simple (Santa Clara, CA, USA) after being incubated on a PVDF membrane with protein as per the Super ECL Enhanced Pico Light Chemiluminescence Kit (SQ 101, Epizyme). The protein expression level was analyzed by ImageJ (National Institutes of Health, USA).

### Absolute Real-Time PCR

RNA isolation and reverse transcription were performed as per the previous study (20). Kidney cDNAs were chosen to prepare SUCNR1 and tubulin standard samples by PCR. After the concentration was detected, the copies per microliter were calculated, and the calibration was set after dilution by 10 multiple gradients. Real-time PCR was carried out via the Applied Biosystems QuantStudio 3 (Thermo Fisher Scientific) to calculate SUCNR1 copies compared with tubulin copies. The primers for SUCNR1 and tubulin were as follows: mouse SUCNR1, 5′-CGAGACAGAAGCCGACAGCA-3′ (sense) and 5′-TAGCCAAACACCACAGTGACAT-3′ (anti-sense); mouse tubulin, 5′-GATCTTCTGGAGCAGTGCGA-3′ (sense) and 5′-GGAGAGATTGACTTACTGGATTGC-3′ (anti-sense); and they were prepared in Tsingke Biotechnology Co., Ltd.

### Biochemical Analysis

Creatine phosphate (CP) and acetyl Co-A concentrations were detected by an enzyme-linked immunosorbent assay (ELISA) kit according to the manufacturer's instructions (Shanghai Ruifan Biological Technology Co., Ltd, Shanghai, China). Similarly, creatine kinase (CK) activity, complex I activity, and complex II activity were quantified by a commercial product of Solarbio Life Science Beijing, China. The NADH/NAD^+^ ratio and ATP content were computed according to Beyotime (China). And the glucose content and reactive oxygen species (ROS) content were detected by kits from Rsbio. All the quantification was performed as per the instructions of the company.

### Muscle Force Measurement

Maximum muscle force was tested by a grip strength meter (YLS-13A, Jinan Yiyan Technology Development Co., Ltd). Briefly, the mouse's tail was grabbed, and the mouse was put on the grip strength meter; after all limbs of the mouse caught the grip strength meter, the mouse was gently pulled to make it resist, and the resist force value was read to analyze.

Weight lifting is another method to measure the muscular strength of mice, and the method was followed as described in the previous study (21). In brief, the middle part of the tail of each mouse was grabbed, and the mouse was then lowered to be able to grasp the different weights. After grasping the weight, the mouse was raised until the weight was also fully raised. If the mouse could hold the weight for 3 s, then it meets the criteria. If not, the time until the weight was dropped was noted, and the trial was repeated after a 10 s rest. Before being able to successfully hold the weight for 3 s, each mouse was allowed to undergo the trial five times. Once the mouse is able to successfully hold the weight for 3 s, it was allowed to try the next heavier weight. The device comprised seven weights, the weights being 26, 36, 46, 66, 86, 106, and 126 g, corresponding to scores of 1, 2, 3, 4, 5, 6, and 7, respectively. The sum of all the scores held by the mouse represents the score of each mouse.

Similarly, the mice performed the treadmill-running test on the FT-200 animal treadmill at an initial speed of 10 m/min for 10 min until mice adaptation. Then, the speed was raised by 1 m/min every 3 min in low-speed running tests.

### Statistical Analysis

All data are presented as means ± the standard error of the mean (SEM). The difference between standard and treated groups was determined by paired *t*-tests; other differences between control and treated groups were determined by unpaired *t*-tests (GraphPad Prism 8.0.1). In the figures, *P* < 0.05 was considered statistically significant (^*^P < 0.05, ^**^P < 0.01, ^***^P < 0.001).

## Results

### SUC Increases Explosive Strength and Oxidative Phosphorylation in Mice

**Figure 1:**
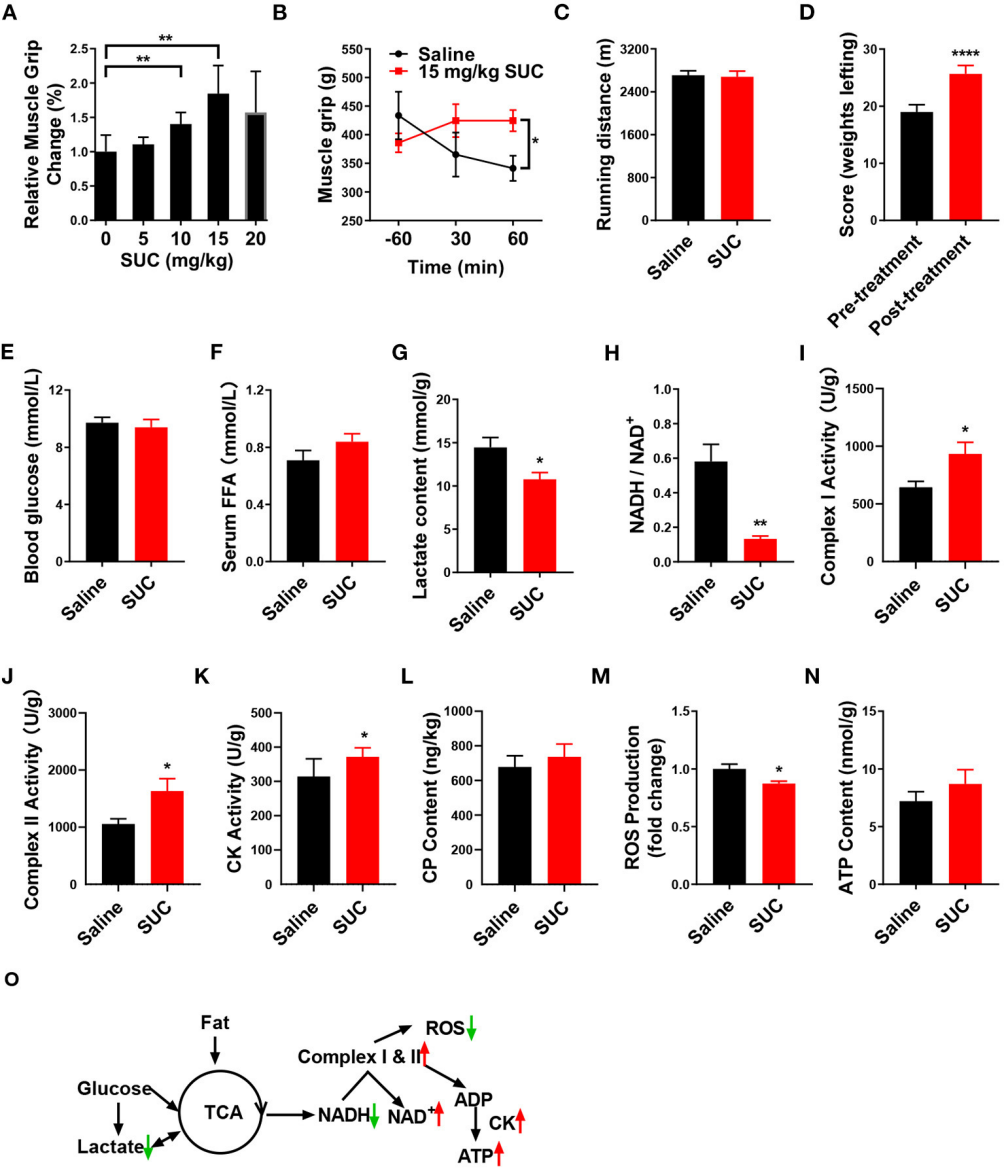
SUC increases explosive strength and mitochondrial oxidative phosphorylation in mice. **(A)** Relative muscle grip of the mice injected with different doses of SUC or saline for 1 h. **(B)** Muscle grip change at different times after injection of 15 mg/kg SUC. **(C)** Running distance of the mice after injecting with saline and SUC for 1 h. **(D)** Weight lifting of the mice after being injected with saline and SUC for 1 h again. Blood and gastrocnemius were collected after the muscle grip test for **(E–N)** results to detect. **(E)** Blood glucose content. **(F)** FFA level in serum. **(G)** Lactate content in muscle. **(H)** NADH/NAD^+^ ratio change in muscle. **(I)** Complex I activity in muscle. **(J)** Complex II activity in muscle. **(K)** Muscle CK activity in muscle. **(L)** Muscle phosphocreatine (CP) content. **(M)** Muscle ROS content. **(N)** Muscle ATP content. **(O)** Schematic diagram of energy production pathway in normal conditions. In the normal process of energy production, acetyl-CoA from glycolysis of glucose and subsequent oxidation of pyruvate and β-oxidation of fatty acid produces NADH by TCA cycle (3). The electrons from NADH pass through the mitochondrial electron transport chain and synthesize ATP by CK in the mitochondrial outer compartment (23). Premature leakage of electrons in the electron transport chain will lead to generating ROS. Lactate, a byproduct of acute exercise, could be used as an energy substrate (24), *n* = 6 mice per group. Each value is shown as the mean ± SEM. *, **, and *** indicate *P* < 0.05, 0.01, and 0.001, respectively.

Our previous study showed that chronic SUC treatment enhanced mice endurance exercise (10). Here, we also found that acute SUC administration could dose-dependently increase muscle grip strength, which was in line with our previous observations. Besides, 15 mg/kg SUC enhanced mice grip strength to the maximum, suggesting that this dose is probably optimal to improve mice explosive strength (Figure 1A). Subsequently, we detected mice grip strength at different time points after 15 mg/kg SUC administration or saline. The result showed that SUC effectively increases this index for 60 min (Figure 1B). Interestingly, our previous evidence confirmed that chronic SUC supplementation significantly increases mice running time and further improves endurance (22), while acute SUC treatment enhanced mice's explosive strength rather than endurance. And so, 15 mg/kg SUC obviously increased mouse weight lifting scores (Figure 1D), but not running distance (Figure 1C).

It is well-established that the body utilizes glucose and fatty acid to provide the energy required for muscle contraction and further maintenance of homeostasis in response to exercise (25, 26). During endurance exercise, the body utilizes fat to produce free fatty acids (FFAs) (27). Here, we found that SUC treatment failed to elevate the serum FFA level (Figure 1F), which indicates that β-oxidation of fatty acid probably does not provide the energy for the enhanced muscle explosive strength by SUC. Oxidative metabolism of glucose produces a large amount of energy under endurance exercise conditions, while glycolysis produces energy rapidly under resistance exercise conditions (28). However, our result showed that 15 mg/kg SUC administration failed to affect mice's blood glucose (Figure 1E), which suggests that increased explosive strength by SUC is independent of glucose metabolism. Notably, SUC treatment markedly decreased the serum lactate level (Figure 1G), indicating that lactate is consumed in resistance exercise after SUC treatment. Importantly, the decreased ratio of NADH/NAD^+^ and the increased activities of mitochondrial complexes I and II indicate that the electron transfer in the mitochondrial respiratory chain was accelerated by short-term SUC treatment (Figures 1H–J). Moreover, SUC treatment failed to affect phosphocreatine (CP) content in the gastrocnemius but increased the CK enzyme activity, which implies that SUC probably could increase energy production (Figures 1K,L).

SUC did not increase the ATP content significantly in skeletal muscle, which prompts us to speculate that ATP produced by SUC probably had been utilized by muscles (Figure 1N). Once oxidative phosphorylation efficiency is reduced, the production of ROS is increased in the mitochondria (29). Decreased ROS content in the SUC group might relate to the enhanced ATP production efficiency (Figure 1M). Taken together, these results indicate that the stimulatory effect of SUC on muscle explosive strength probably requires energy supplement by oxidative phosphorylation rather than fatty acid or glucose energy substrates.

### SUC Promotes Oxidative Phosphorylation in C2C12 Cells

**Figure 2:**
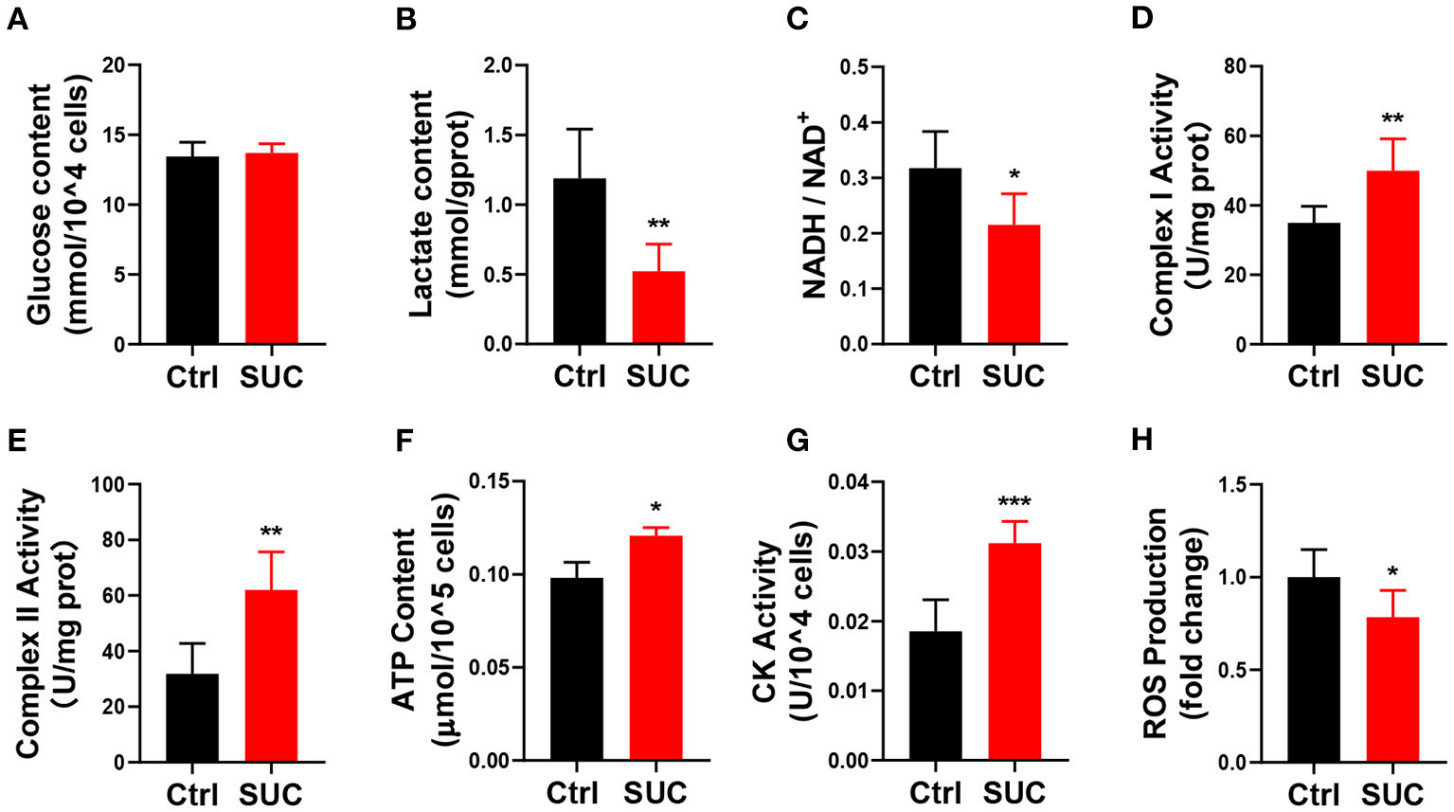
SUC promotes oxidative phosphorylation in C2C12 cells. C2C12 cells could be observed in mature fibers after induced differentiation for 4–6 days, and then cells were treated with 1 mM SUC for 1 h to detect oxidative phosphorylation. **(A)** Glucose content in cells. **(B)** Lactate content. **(C)** NADH/NAD^+^ ratio change. **(D)** Complex I activity. **(E)** Complex II activity. **(F)** ATP content. **(G)** CK activity. **(H)** ROS fold change compared to the control group. *n* = 6 for each group. Each value is shown as the mean ± SEM. *, **, and *** indicate *P* < 0.05, 0.01, and 0.001, respectively.

We next examined if *in vitro* SUC treatment would produce similar oxidative phosphorylation effects, as we observed in mice receiving acute SUC administration. Here, we characterized SUC's effects on C2C12 cells by following the previous study (30). Consistent with observations *in vivo*, 1 mM SUC treatment effectively decreased the lactate content, NADH/NAD^+^ ratio, and ROS content (Figures 2B,C,H) and increased the mitochondrial complex I and II activity, ATP content, CK activity (Figures 2D–G). However, it failed to affect glucose content in C2C12 cells (Figure 2A). These data support the view that SUC enhances oxidative phosphorylation in C2C12 cells.

### SUCNR1 Is Required for the Short-Term Effects of SUC in C2C12 Cells

**Figure 3:**
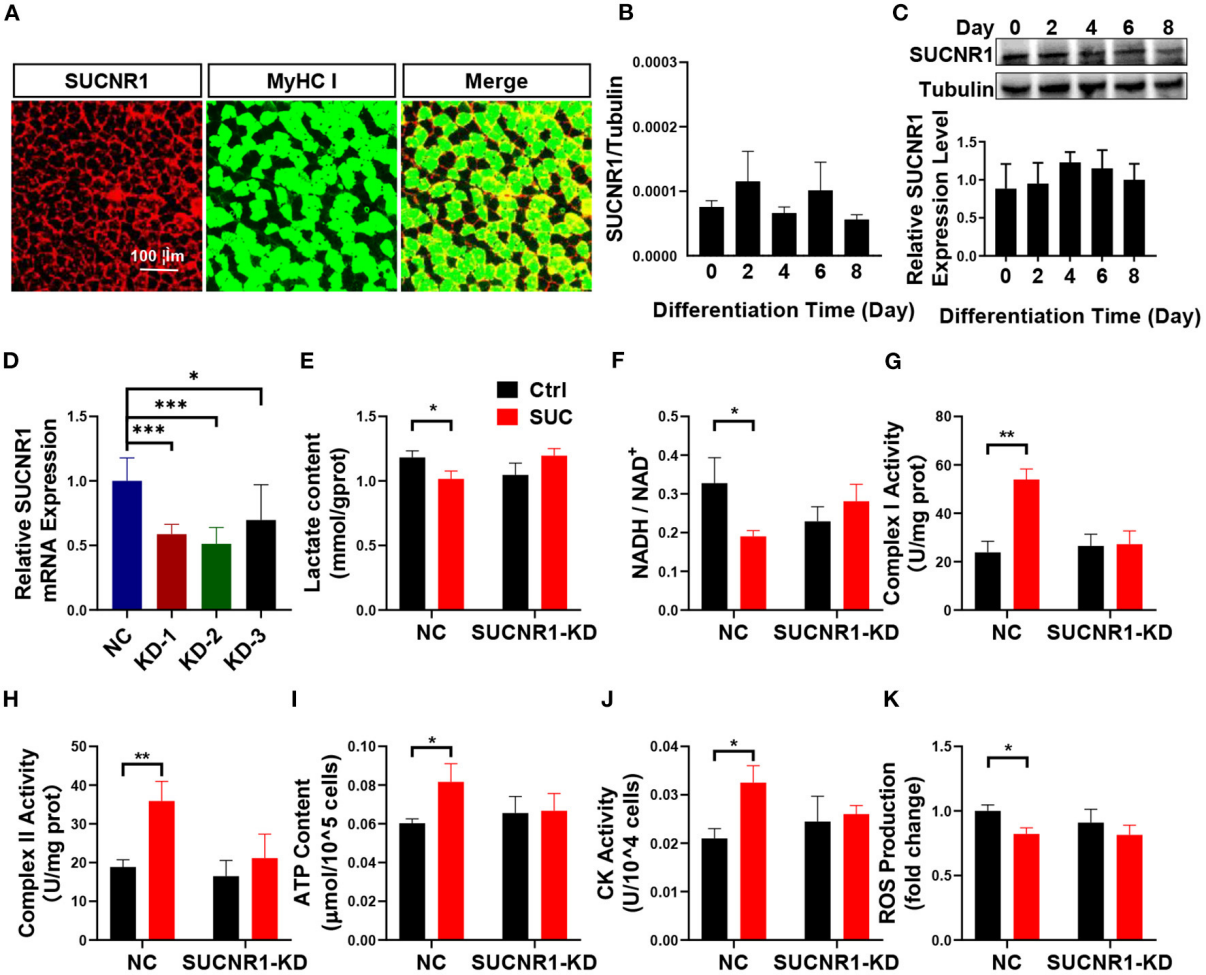
SUCNR1 is required for the short-term effects of SUC in C2C12 cells. **(A)** Immunofluorescence staining; red for SUCNR1 and green for MyHC I. **(B)** Absolute quantitative detection of SUCNR1 mRNA expression level compared to tubulin. **(C)** WB to detect the SUCNR1 protein expression level in a different induced day. **(D)** SUCNR1 relative mRNA expression level after being treated with three different SUCNR1 siRNAs for 24 h. The cells in **(E–K)** SUCNR1 knockdown by SUCNR1 siRNA and were then induced to differentiate for 4–6 days; 1 mM SUC cells were treated for 1 h to detect related factors. **(E)** Lactate content. **(F)** NADH/NAD^+^ ratio change. **(G)** Complex I activity. **(H)** Complex II activity. **(I)** ATP content. **(J)** CK activity. **(K)** ROS fold change compared to ctrl group. *n* = 6 per group. Each value is showed as the mean ± SEM. *, **, and *** indicate *P* < 0.05, 0.01, and 0.001, respectively.

To explore the mechanism in which SUC increased oxidative phosphorylation in skeletal muscle, we characterized the effect of the SUC membrane receptor SUCNR1 on SUC-induced oxidative phosphorylation. SUC exhibits many functions through SUCNR1, but the existence of SUCNR1 in muscles cells is controversial (18). The IF result showed that SUCNR1 is enriched around the muscle fiber membrane (Figure 3A). Similarly, absolutely quantitative PCR proved the existence of SUCNR1 mRNA in differentiated C2C12 cells (Figure 3B). Furthermore, the results of WB showed that the SUCNR1 protein was expressed in differentiated C2C12 cells (Figure 3C).

These results suggest a possible role of SUCNR1 in stimulatory effects of SUC on oxidative phosphorylation. To test this point, we first generated a loss-of-function C2C12 cell model by using siRNA to target SUCNR1 specifically. We found that SUCNR1 siRNA-treated C2C12 cells showed significantly less mRNA expression of SUCNR1 compared to control siRNA-treated cells (Figure 3D), which validated our SUCNR1-knockdown C2C12 cell model. By using this model, we showed that the knockdown of SUCNR1 abolished the stimulatory effects of SUC on the mitochondrial complex I and II activity, ATP content, and CK activity (Figures 3G–J) and reversed the inhibitory effects of SUC on the lactate content, NADH/NAD^+^ ratio, and ROS content (Figures 3E,F,K). These results supported an intermedia role of SUCNR1 in SUC-induced oxidative phosphorylation.

### SUCNR1 Knockout Reversed the Effect of SUC on Explosive Strength

**Figure 4:**
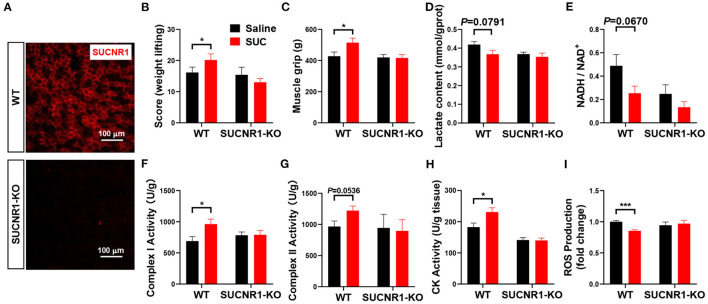
SUCNR1 knockout reversed the effect of SUC on explosive strength. Mice were injected with saline or 15 mg/kg SUC for 1 h to detect the muscle force, and then gastrocnemius were collected for the following detection. **(A)** IF stains red for SUCNR1. **(B)** Weight lifting and **(C)** muscle grip to detect the mouse explosive strength. **(D)** Lactate content. **(E)** NADH/NAD^+^ ratio change. **(F)** Complex I activity. **(G)** Complex II activity. **(H)** CK activity in muscle. **(I)** ROS production fold change. Each group *n* = 6 mice. Each value is shown as the mean ± SEM. *Indicate *P* < 0.05, ***Indicate *P* < 0.001.

To further determine the role of SUCNR1 in SUC metabolic effect *in vivo*, we generated the SUCNR1 global knockout mouse model (SUCNR1-KO) by using the Clustered Regularly Interspaced Short Palindromic Repeats (CRISPR) method (10). We found that SUCNR1-KO mice completely abolished SUCNR1 expression compared to WT mice (Figure 4A), which validates that our knockout model could be used in the following experiments. Consistent with the *in vitro* C2C12 cell model, SUCNR1 knockout abolished SUC-induced effects, indicated by increasing weight lifting, muscle grip, complex I and II activity, and CK activity (Figures 4B,C,F,G,H). Additionally, SUCNR1 deletion also eliminated SUC-induced inhibitory effects on the lactate content, NADH/NAD^+^ ratio, and ROS content (Figures 4D,E,I). These results illustrated that SUC accelerated electron transfer in the respiratory chain through SUCNR1. The enhancement of the CK activity effect indicates that the SUC promoted oxidative phosphorylation by SUCNR1. Thus, the results from both loss-of-function models demonstrate that SUCNR1 mediates the SUC administration-induced increase of explosive strength and oxidative phosphorylation.

## Discussion

Explosive strength and endurance training play an important role in animal exercise capacity and body health (31), distinguished by different physical performance characteristics, energy resources, and energy production processes (32). In general, the regents enhance explosive power with lower endurance exercise, and vice versa. SUC is an intermediate in the TCA cycle, and a previous study has found that long-term feeding of SUC can transform the fast muscle fiber types to slow muscle fiber types and enhance the endurance exercise of mice (10). Interestingly, we found that the acute administration of SUC enhanced the explosive strength of mice. Many factors could influence explosive strength, such as the distribution of nerve synapses, the ratio of muscle fiber types, blood flow, and metabolism (33–36). Acute effects make it hard to increase the distribution of nerve synapses or the proportion of fast muscle fibers, and the change in blood flow is often accompanied by the consumption of blood glucose (37). However, the treatment of SUC did not affect the blood glucose. Therefore, acute administration of SUC is more likely to change energy metabolism to enhance explosive strength in skeletal muscle. In normal conditions, the energy source of explosive strength is mainly produced through glycolysis and β-hydrolysis of fatty acid to produce ATP rapidly, with a large amount of lactate generation simultaneously (38). Our results exhibited that the blood glucose and serum FFA content did not change by acute SUC administration, but the lactate content in skeletal muscle was significantly reduced. Therefore, these results demonstrated that the enhanced explosive power of SUC does not rely on glycolysis and β-oxidation of fatty acids.

With the treatment of SUC, ATP was increased by enhancing electron transfer efficiency and oxidative phosphorylation. In physiological conditions, the mitochondrial electron transport chain transits electrons through systematic electron transfer reactions and generates ATP for energy supply through the coupling effect (24, 39). ROS is produced by the premature leak of electrons from the mitochondrial electron transport chain, which will lead to the decrease in the potential energy of electrons used to synthesize ATP (40, 41). Therefore, the accumulation of ROS is often accompanied by inefficient ATP synthesis (42). Our results found that acute SUC treatment notably decreased mice ROS content in skeletal muscle and C2C12 cells, which suggested that the energy metabolism efficiency induced by SUC is increased. Here, our results revealed that SUC intervention decreased the NADH/NAD^+^ ratio, enhanced mitochondrial complex enzymes, increased CK activity, and increased skeletal muscle strength caused by increased ATP production. These results indicate that oxidative phosphorylation was increased by SUC acute treatment (23).

SUC has many biological functions with various mechanisms. Intracellular SUC can be oxidized to fumarate under the catalysis of mitochondrial complex II, which participates in energy metabolism (43, 44) and affects epigenetics through SUC dehydrogenase (45). Even though SUC is not permeable to mammalian cell membranes (11, 13, 14), it can activate calcium ion signals via the membrane receptor SUCNR1 (22). Some people held the opinion that SUCNR1 was not expressed in skeletal muscle cells (18), but we found that SUCNR1 mRNA and protein were expressed on C2C12 cells, and the fluorescence of SUCNR1 presented around the skeletal muscle fibers, which is consistent with some research findings (17). The mitochondrial calcium level strictly controls mitochondrial ATP production (46), which could stimulate complex III and ATP synthase on the electron transport chain to accelerate oxidative phosphorylation (47). We have proved that SUC can activate calcium signals after activating SUCNR1 in our previous study (10). Consequently, we conjectured that SUC could increase energy production due to the effect of SUC on the mitochondrial calcium signal by increasing intracellular calcium.

Altogether SUC enhances the energy production efficiency by consuming lactate consumption and accelerating oxidative phosphorylation via SUCNR1 under the condition of rapid exercise.

## Conclusion

In conclusion, the acute administration of SUC enhanced mitochondrial oxidative phosphorylation, accelerated electron transfer in the mitochondrial oxidative respiratory chain, greatly increased energy for acute exercise, and consumed lactate. The interaction mechanism of SUCNR1 activation with mitochondrial oxidative phosphorylation should be further developed.

## Data Availability Statement

The original contributions presented in the study are included in the article/supplementary material, further inquiries can be directed to the corresponding authors.

## Ethics Statement

The animal study was reviewed and approved by Animal Subjects Committee of South China Agricultural University.

## Author Contributions

Data acquisition and methodology were completed by GX and GS was the main contributor to the study conceptualization, article reviewing, and editing. GX, YY, and PL contributed to the manuscript writing. JY and JZ supported mice feeding. All authors were involved in data analysis and data interpretation.

## Funding

This study was supported by the research project of Guangdong Laboratory for Lingnan Modern Agriculture (NZ2021028); the local innovative and research team project of Guangdong province (2019BT02N630); Guangdong Key Research and Development Program (2019B020218001).

## Conflict of Interest

The authors declare that the research was conducted in the absence of any commercial or financial relationships that could be construed as a potential conflict of interest.

## Publisher's Note

All claims expressed in this article are solely those of the authors and do not necessarily represent those of their affiliated organizations, or those of the publisher, the editors and the reviewers. Any product that may be evaluated in this article, or claim that may be made by its manufacturer, is not guaranteed or endorsed by the publisher.
